# Prediction of Functional Outcomes at Discharge Using Plasma Concentration of von Willebrand Factor Antigen at Admission in Hospitalized Patients with COVID-19

**DOI:** 10.3400/avd.oa.25-00153

**Published:** 2026-02-05

**Authors:** Masayuki Oki, Daisuke Yamasawa, Shinichi Goto, Hidetaka Yanagi, Saki Manabe, Takako Kobayashi, Ayumi Tsuda, Shota Sato, Seiji Morita, Yoshihide Nakagawa, Tsuyoshi Oguma, Koichiro Asano, Norio Yamamoto, Hideki Ozawa, Shinya Goto

**Affiliations:** 1 Division of General Internal Medicine and Family Medicine, Department of General and Acute Medicine, Tokai University School of Medicine, Isehara, Kanagawa, Japan; 2 Department of Emergency and Critical Care Medicine, Tokai University School of Medicine, Isehara, Kanagawa, Japan; 3 Division of Pulmonary Medicine, Department of Internal Medicine, Tokai University School of Medicine, Isehara, Kanagawa, Japan; 4 Department of Microbiology, Tokai University School of Medicine, Isehara, Kanagawa, Japan; 5 Department of Medicine (Cardiology), Tokai University School of Medicine, Isehara, Kanagawa, Japan

**Keywords:** COVID-19, von Willebrand factor, ADAMTS13, endothelial injury, functional outcome

## Abstract

**Objectives:**

Coronavirus disease 2019 (COVID-19) causes endothelial injury through inflammatory and hypoxic stress, leading to vascular dysfunction and immunothrombosis. The plasma level of von Willebrand factor (VWF) could serve as a biomarker of vascular injury. While elevated VWF predicts mortality in severe COVID-19, its relationship with post-discharge functional outcomes remains unclear. This study aimed to determine whether plasma VWF antigen (VWF:Ag) levels at admission predict functional status at discharge in patients hospitalized for COVID-19.

**Methods:**

This was a single-center prospective cohort study conducted at Tokai University Hospital from July to September 2021. We evaluated the relationship between plasma VWF:Ag levels at admission and a Clinical Frailty Scale (CFS) score ≥4 at discharge using univariable and multivariable logistic regression analyses.

**Results:**

A total of 97 patients were enrolled in the study. The median VWF:Ag level at admission was 330.0% (95% confidence interval [CI]: 273.0–391.8). Univariable analysis showed a significant association between elevated VWF:Ag levels and CFS score ≥4 at discharge. This association remained significant after adjusting for age and sex (odds ratio 1.010, 95% CI: 1.000–1.010, p = 0.005).

**Conclusion:**

Elevated VWF:Ag levels at admission predict poor functional outcomes at discharge in COVID-19 patients, independent of age and sex.

## Introduction

The severe acute respiratory syndrome coronavirus 2 (SARS-CoV-2) virus attacks both airway epithelial cells and vascular endothelial cells.^1–3)^ Damage to airway epithelial cells leads to severe lung injury,^4)^ while damage to vascular endothelial cells results in extensive vascular disorders.^4)^ The prognosis is affected by the severity of both types of injuries. Plasma biomarkers of endothelial injury can provide insights into the extent of vascular damage at the time of admission.^5,6)^

Von Willebrand factor (VWF) is primarily released from damaged vascular endothelial cells.^7)^ Plasma VWF serves as a biomarker of endothelial dysfunction in cardiovascular diseases.^8–10)^ In coronavirus disease 2019 (COVID-19), SARS-CoV-2 infects endothelial cells via the angiotensin-converting enzyme (ACE2) receptor,^4,11)^ triggering immunothrombosis—a key pathophysiologic mechanism.^12)^ While elevated plasma VWF antigen (VWF:Ag) levels predict in-hospital mortality in severe COVID-19,^13,14)^ their ability to predict functional recovery and discharge status remains unclear. Given that endothelial injury may influence the post-acute phase of functional capacity, we hypothesized that VWF:Ag levels at admission predict impaired functional status at discharge. This study aimed to investigate whether plasma VWF:Ag levels measured at hospital admission predict functional outcomes at discharge in hospitalized COVID-19 patients.

## Materials and Methods

### Study population

All consecutive patients admitted to Tokai University Hospital for SARS-CoV-2 infection between July and September 2021 were recruited for this study. The Institutional Review Board approved the study (21R058), and written informed consent was obtained from all participants.

### Clinical outcome of interest

The primary outcome measured was impaired functional status at discharge, which was defined as a Clinical Frailty Scale (CFS) score of 4 or higher.^15,16)^ We used the 7-point CFS scale (scores 1–7).^17)^ In the original 7-point CFS, score 7 encompasses both “severely frail” patients and those who are “terminally ill” or approaching death.^15)^ For the purpose of this analysis, we focused exclusively on patients who were discharged alive, as the CFS is designed primarily to assess functional status. Therefore, the 8 patients who died during hospitalization were excluded from the primary analysis comparing CFS score ≥4 versus CFS score <4. The in-hospital mortality rate (8.2%) is reported separately as a clinical outcome.

### Measured parameters

The following plasma parameters were measured at admission and serial intervals: VWF:Ag levels, VWF cleavage protease ADAMTS13 (a disintegrin and metalloproteinase with thrombospondin type 1 motifs member) levels, and other parameters, including blood cell counts, inflammation markers, and thrombosis markers.

### Primary hypothesis and statistical analysis

We hypothesized that plasma VWF:Ag levels at admission could predict an individual’s functional status at discharge. To investigate the association between VWF:Ag levels at admission and a CFS score of 4 or higher at discharge, we used both univariable and multivariable logistic regression analyses, adjusting for age and sex. A 2-sided p-value of less than 0.05 was considered statistically significant. Patients who died during hospitalization (n = 8) were excluded from the logistic regression analysis, as the CFS scale is designed to assess functional status in living patients rather than mortality outcomes.

## Results

### Baseline characteristics

A total of 97 patients were recruited for the study. Eight patients (8.2%) died during hospitalization and were excluded from the primary analysis of functional outcomes at discharge. The remaining 89 patients who were discharged alive were included in the analysis comparing CFS scores <4 (n = 60) versus ≥4 (n = 29). Many of the patients were male, accounting for 73.2% of the cohort, and the median age was 56 years, with an interquartile range (IQR) of 49–62 years. The risk factor profile among the patients was generally low. A limited smoking history was noted, with 22.6% classified as former smokers and 16.1% as current smokers. The prevalence of obesity was low at 28.9%, along with a history of hypertension (35.4%), cardiovascular diseases (5.2%), diabetes mellitus (30.9%), active cancer (3.1%), and chronic lung diseases (10.3%) (**Table 1**).

**Table 1:** Patient characteristics and parameters measured at hospital admission

Factor	Group	N (%) or median [IQR]
n		97
Age		56.00 [49.00, 62.00]
Gender	Male	71 (73.2)
	Female	26 (26.8)
Smoking history	Current	15 (16.1)
	Former	21 (22.6)
	No	57 (61.3)
Drinking history	Yes	34 (37.4)
	No	57 (62.6)
Obesity (BMI >30)	Yes	28 (28.9)
	No	69 (71.1)
Hypertension	Yes	34 (35.4)
	No	62 (64.6)
Cardiovascular disease	Yes	5 (5.2)
	No	92 (94.8)
Diabetes mellitus	Yes	30 (30.9)
	No	67 (69.1)
Active cancer	Yes	3 (3.1)
	No	94 (96.9)
Chronic lung disease	Yes	10 (10.3)
	No	87 (89.7)
CKD	Yes	7 (7.2)
	No	90 (92.8)
Immunocompromised hosts	Yes	4 (4.1)
	No	93 (95.9)
Liver cirrhosis	Yes	1 (1.0)
	No	96 (99.0)
VWF-related		
VWF:Ag (%)		330.0 [273.0, 391.8]
ADAMTS13 activity (%)		74.0 [64.0, 80.0]
VWF/ADAMTS13 ratio		4.3 [3.6, 5.8]
Inflamation-related		
CRP (mg/dL)		8.5 [2.7, 13.5]
WBC (/μL)		7900.0 [5400.0, 11100.0]
Absolute lymphocyte counts (/μL)		623.4 [403.5, 844.5]
Thrombosis-related		
D-dimer (μg/mL)		1.0 [0.6, 1.8]
PAI-1 (ng/mL)		56.0 [44.8, 76.5]
TM (U/mL)		22.8 [15.7, 26.3]
Others		
Albumin (g/dL)		2.9 [2.8, 3.1]
CPK (U/L)		130.5 [54.0, 228.5]
LD (U/L)		526.0 [415.0, 654.0]
Creatinine (mg/dL)		0.8 [0.7, 1.1]
Troponin T (ng/mL)		6.0 [4.0, 22.0]
Ferritin (ng/mL)		1251 [604, 2098]

The values in brackets indicate the 25th and 75th percentiles, respectively.

BMI: body mass index (kg/m^2^); CKD: chronic kidney disease; VWF:Ag: von Willebrand factor:antigen; ADAMTS13: a disintegrin and metalloproteinase with thrombospondin type 1 motifs member; CRP: C-reactive protein; WBC: white blood cell counts; PAI-1: plasminogen activator inhibitor-1; TM: thrombomodulin; CPK: creatine phosphokinase; LD: lactate dehydrogenase; IQR: interquartile range

### Plasma markers at admission

Patients exhibited significant inflammation, as evidenced by an elevated C-reactive protein (CRP) level with a median of 8.5 mg/dL (95% confidence interval [CI]: 2.7–13.5). White blood cell (WBC) counts were nearly normal, averaging 7900/μL (95% CI: 5400–11100). Additionally, D-dimer levels were elevated at 1.0 μg/mL (95% CI: 0.6–1.8). The VWF:Ag level was notably high at 330.0% (95% CI: 273.0–391.8), while the ADAMTS13 level was lower, recorded at 74.0% (95% CI: 64.0–80.0) (**Table 1**).

### Clinical course

High-flow oxygen therapy was utilized in 59.8% of cases, while 24.7% of patients required mechanical ventilation. Hemodialysis and extracorporeal membrane oxygenation (ECMO) were necessary for 6.2% of the patients each. During hospitalization, 8 patients (8.2%) passed away. Of those who were discharged, 37.1% returned home, while 62.9% were transferred to secondary hospitals or nursing homes (**Table 2**).

**Table 2:** Clinical course of admitted patients

Prone position	
Yes	69 (74.2)
No	24 (25.8)
High-flow oxygen therapy	
Yes	58 (59.8)
No	39 (40.2)
Ventilator	
Yes	24 (24.7)
No	73 (75.3)
Vasopressors	
Yes	19 (19.6)
No	78 (80.4)
Hemodialysis	
Yes	6 (6.2)
No	91 (93.8)
ECMO	
Yes	6 (6.2)
No	91 (93.8)
CFS score at discharge	
1	2 (2.1)
2	24 (24.7)
3	34 (35.1)
4	6 (6.2)
5	7 (7.2)
6	10 (10.3)
7	14 (14.4)
Death during hospitalization	
Yes	8 (8.2)
No	89 (91.8)
Discharge destination	
Home	33 (37.1)
(If alive at discharge)	
Transfer	56 (62.9)

CFS (7-point scale, scores 1–7) assessed among patients discharged alive. Score 1 = very fit, 4 = vulnerable/living with limited activity, 7 = severely frail, including those approaching death. The 8 patients who died during hospitalization were excluded from CFS categorization and are reported separately under “Death during hospitalization.”

ECMO: extracorporeal membrane oxygenation; CFS: Clinical Frailty Scale

**Fig. 1:**
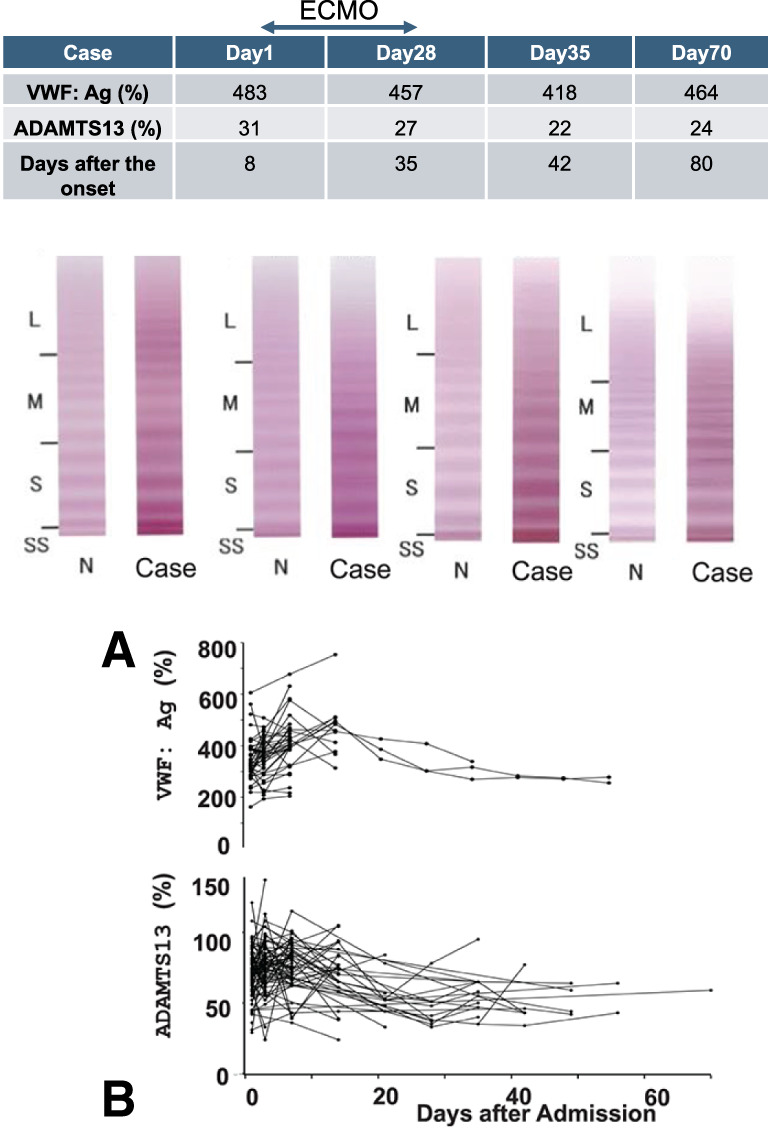
(**A**) A representative Western blot analysis of VWF multimers. It indicates that VWF levels are elevated, yet there are no significant differences in the distribution of multimers when compared to standard plasma. (**B**) The changes in VWF and ADAMTS13 levels over the course of admission, showing consistently low levels of ADAMTS13 alongside high levels of VWF. VWF:Ag: von Willebrand factor:antigen; ECMO: extracorporeal membrane oxygenation; ADAMTS13: a disintegrin and metalloproteinase with thrombospondin type 1 motifs member

A representative Western blot analysis of VWF multimers indicates elevated levels of VWF:Ag. However, there are no significant differences in the distribution of multimers when compared to standard plasma (**Fig. 1A**). Furthermore, the changes in VWF:Ag and ADAMTS13 levels during the admission period reveal consistently low levels of ADAMTS13 alongside high levels of VWF:Ag (**Fig. 1B**).

### Functional status at discharge

Among the 89 patients who were discharged alive, 29 had a CFS score of 4 or higher. At baseline, no clinical characteristics were significantly associated with a CFS score of 4 or higher, except for gender (p = 0.037) (**Table 3**). Several plasma markers showed a correlation with a CFS of 4 or higher (**Table 3**): lower albumin levels (p = 0.02), higher D-dimer levels (p < 0.001), elevated interleukin-6 (IL-6) levels (p = 0.006), increased lactate dehydrogenase (LD) (p = 0.005), higher VWF:Ag levels (p = 0.001), and a higher VWF/ADAMTS13 ratio (p = 0.001).

**Table 3:** Baseline characteristics and laboratory parameters by discharge functional status (CFS <4 vs. CFS ≥4)

Factor	Group	CFS <4	CFS ≥4	p-Value
		N (%) or median [IQR]^*^	N (%) or median [IQR]^*^	
N = 89		60	29	
Age		56.0 [49.0, 62.3]	52.0 [49.0, 62.0]	0.927
Gender	Female	11 (18.3)	12 (41.4)	0.037
	Male	49 (81.7)	17 (58.6)	
Smoking history	Current	11 (18.6)	4 (15.4)	0.760
	Former	15 (25.4)	5 (19.2)	
	No	33 (55.9)	17 (65.4)	
Drinking history	Yes	27 (45.8)	6 (25.0)	0.090
	No	32 (54.2)	18 (75.0)	
Obesity (BMI >30)	Yes	15 (25.0)	11 (37.9)	0.224
	No	45 (75.0)	18 (62.1)	
Hypertension	Yes	19 (32.2)	12 (41.4)	0.478
	No	40 (67.8)	17 (58.6)	
Cardiovascular disease	Yes	2 (3.3)	0 (0.0)	1
	No	58 (96.7)	16 (100.0)	
Active cancer	Yes	3 (5.0)	0 (0.0)	0.548
	No	57 (95.0)	16 (100.0)	
Chronic lung disease	Yes	7 (11.7)	3 (10.3)	1
	No	53 (88.3)	26 (89.7)	
CKD	Yes	3 (5.0)	1 (3.4)	1
	No	57 (95.0)	28 (96.6)	
Diabetes mellitus	Yes	16 (26.7)	9 (31.0)	0.802
	No	44 (73.3)	20 (69.0)	
Immunocompromised hosts	Yes	3 (5.0)	1 (3.4)	1
	No	57 (95.0)	28 (96.6)	
Liver cirrhosis	Yes	13 (22.4)	0 (0.0)	0.326
	No	45 (77.6)	16 (100.0)	
Prone position	Yes	53 (74.6)	17 (65.4)	0.760
	No	18 (25.4)	9 (34.6)	
WBC (/μL)		7550.00 [4725.00, 9725.00]	7900.00 [5600.00, 12300.00]	0.261
Albumin (g/dL)		3.00 [2.85, 3.30]	2.90 [2.70, 3.10]	0.02
Creatinine (mg/dL)		0.79 [0.66, 0.95]	0.84 [0.68, 1.20]	0.338
CRP (mg/dL)		7.34 [2.77, 15.04]	4.33 [1.95,12.61]	0.549
D-dimer (μg/mL)		0.70 [0.50,1.10]	1.50 [0.90, 2.70]	<0.001
Ferritin (ng/mL)		3.00 [1.00, 6.00]	2.00 [2.00, 4.00]	0.873
IL-6 (pg/mL)		23.40 [7.83, 54.85]	66.75 [34.77, 229.25]	0.006
LD (U/L)		473.00 [359.25, 583.50]	567.00 [485.00, 777.00]	0.005
Procalcitonin (ng/mL)		0.12 [0.06, 0.24]	0.22 [0.08, 0.45]	0.12
VWF:Ag (%)		301.00 [244.00, 362.00]	391.00 [326.00, 412.00]	<0.001
ADAMTS13 activity (%)		73.50 [66.75, 80.00]	71.00 [62.00, 81.00]	0.566
VWF/ADAMTS13		4.10 [3.21, 4.98]	5.08 [4.32, 6.59]	0.001
PAI-1 (ng/mL)		53.00 [43.50, 68.25]	61.00 [46.25, 103.50]	0.07
TM (U/mL)		18.15 [14.80, 24.35]	20.90 [17.50, 27.10]	0.078

* The values in brackets indicate the 25th and 75th percentiles, respectively.

BMI: body mass index (kg/m^2^); CKD: chronic kidney disease; WBC: white blood cell counts; CRP: C-reactive protein; IL-6: interleukin-6; LD: lactate dehydrogenase; VWF:Ag: von Willebrand factor:antigen; ADAMTS13: a disintegrin and metalloproteinase with thrombospondin type 1 motifs member; PAI-1: plasminogen activator inhibitor-1; TM: thrombomodulin; CFS: Clinical Frailty Scale; IQR: interquartile range

### Prediction of functional outcome

The univariable analysis indicated that both VWF:Ag levels and the VWF/ADAMTS13 ratio were significantly associated with a CFS score of 4 or higher. This significance remained after adjusting for age and sex (**Table 4**). The adjusted odds ratio (OR) for VWF:Ag levels was 1.010 (95% CI: 1.000–1.010, p = 0.005), while the adjusted OR for the VWF/ADAMTS13 ratio was 1.360 (95% CI: 1.070–1.730, p = 0.013).

**Table 4:** Adjusted odds ratios for moderately impaired functional outcome (CFS ≥4) at discharge in COVID-19 patients

Variable	Adjusted OR	95% CI	p-Value
VWF:Ag (%)	1.010	1.000–1.010	0.005
ADAMTS13 (%)	0.989	0.963–1.020	0.435
VWF/ADAMTS13 ratio	1.360	1.070–1.730	0.013
TM (U/mL)	1.030	0.990–1.070	0.142

Results are adjusted for age and sex.

OR: odds ratio; CI: confidence interval; CFS: Clinical Frailty Scale; VWF:Ag: von Willebrand factor:antigen; ADAMTS13: a disintegrin and metalloproteinase with thrombospondin type 1 motifs member; TM: thrombomodulin; COVID-19: coronavirus disease 2019

## Discussion

Our single-center study confirmed that plasma levels of VWF:Ag were significantly elevated at admission, which predicted poor functional outcomes at discharge represented by CFS. This suggests that the extent of vascular damage has prognostic value. Larger VWF multimers are cleaved by ADAMTS13,^18)^ and decreased levels of ADAMTS13 at admission indicate its consumption during the cleavage of VWF from virus-stimulated endothelial cells.^19)^ The VWF/ADAMTS13 ratio, a marker of the balance between thrombosis and bleeding,^20)^ was elevated and predicted poor outcomes.

CFS is widely used to determine the need for admission to a decision unit or intensive care unit (ICU) globally.^17)^ National Institute for Health and Care Excellence guidelines for critical care also recommend the use of CFS for selecting patients suitable for ICU admission.^21)^ In Japan, CFS was used also for “download transfer” of recovered patients from high-intensity hospitals to the lower-grade ones. Thus, the CFS is one of the most widely used scales for evaluating the recovery status of patients admitted to high-intensity hospitals such as Tokai University Hospital. Obviously, the CFS scale is influenced largely by time course, especially in patients with infectious diseases, including COVID-19. However, during the COVID-19 pandemic, the Japanese government guided the prompt “download transfer” of severe COVID-19 patients from high-intensity hospitals to lower-intensity hospitals after recovery. Thus, it was practically difficult to measure CFS with a fixed period. Given the importance of time as an influencing factor for CFS, measuring CFS at discharge may pose a significant limitation. Moreover, the CFS is a rather general parameter that reflects the functional state of the target patients.^22)^ It reflects the severity of thrombosis or bleeding, as well as the simple damage caused by viral infection. Our study provides prognostic value for VWF:Ag level at admission. However, the causal link between increased VWF:Ag at admission and the poor CFS at discharge is still to be elucidated.

Regarding our choice of scale, we utilized the 7-point CFS (scores 1–7) rather than the 9-point version. When this study was planned in 2021, the 7-point scale was familiar to our team and was the standard tool in our institution. In the original 7-point scale, score 7 encompasses both severely frail patients and those approaching death, acknowledging that these states are often indistinguishable in acute illness.^15)^ During the download transfer process, we faced significant challenges differentiating between severe frailty and impending mortality in the long-term phase of COVID-19. Patients often exhibited profound functional impairment that made it impossible to reliably predict short-term mortality at the time of transfer decisions.

The 7-point scale was therefore pragmatically suited to our clinical context. Because the CFS is fundamentally a tool for assessing functional status rather than predicting mortality, we excluded the 8 patients who died from the functional analysis. This ensures that the relationship between VWF:Ag and recovery of functional capacity is assessed clearly among survivors.

Our study involved a relatively young population, approximately 10% of whom experienced mortality. Previous reports indicate that hospitalized deaths occur mainly in older patients^23)^ with cardiovascular disease and a history of chronic kidney disease (CKD).^24)^ Additionally, high-flow oxygen therapy or noninvasive ventilation (World Health Organization [WHO] scale 4) increases the risk of mortality.^25)^ Our findings are consistent with the published literature. Deceased patients showed lower lymphocyte counts^26)^ and serum albumin,^27)^ while exhibiting elevated D-dimer,^28)^ lactate dehydrogenase (LD),^29)^ interleukin-6 (IL-6),^30)^ and thrombomodulin (TM).^31)^ These results confirm that undernutrition indicated by lower albumin levels and extensive endothelial damage indicated by increased D-dimer, (LD), (IL-6), and TM are associated with worse outcomes.

Baseline levels of VWF:Ag and the VWF/ADAMTS13 ratio can predict functional outcomes at discharge. VWF is secreted from endothelial cells, megakaryocytes, or platelets.^32)^ Endothelial injuries during SARS-CoV-2 infection are highlighted as an indirect consequence caused by inflammatory signaling and hypoxic stress from infected epithelial cells, which leads to vascular dysfunction and immunothrombosis.^2)^ Platelet adhesion triggers the degranulation of α-granules, leading to VWF release.^33)^ Activated platelet-derived VWF contributes approximately 20% of total plasma VWF; both endothelial and platelet-derived VWF are responsible for elevated levels seen in SARS-CoV-2 infections. High VWF:Ag levels indicate significant endothelial damage, which aligns with clinical understanding.

### Limitations

This single-center registry may limit the generalizability. However, the similarity in baseline characteristics and blood markers to previous reports suggests that our population did not differ substantially.

## Conclusion

Levels of VWF:Ag and VWF/ADAMTS13 measured upon admission may help predict the functional status of hospitalized COVID-19 patients at discharge.
